# Cholecystolithiasis Is Associated with *Clonorchis sinensis* Infection

**DOI:** 10.1371/journal.pone.0042471

**Published:** 2012-08-08

**Authors:** Tie Qiao, Rui-hong Ma, Xiao-bing Luo, Zhen-liang Luo, Pei-ming Zheng

**Affiliations:** 1 Laboratory of Gallbladder Diseases, Institute of Gallbladder Disease of Panyu, Guangzhou, People’s Republic of China; 2 Laboratory of Gallbladder Diseases, The Second People’s Hospital of Panyu, Panyu, Guangzhou, People’s Republic of China; Instituto de Higiene e Medicina Tropical, Portugal

## Abstract

**Background:**

The objective of this study was to analyze gallbladder stones for direct evidence of a relationship between *Clonorchis sinensis* infection and gallbladder stones formation.

**Methodology:**

We investigated one hundred eighty-three gallbladder stones for the presence of *Clonorchis sinensis eggs* using microscopy, and analyzed their composition using Fourier transform infrared spectroscopy. We confirmed the presence of *Clonorchis sinensis* eggs in the gallbladder stones using real-time fluorescent PCR and scanning electron microscopy.

**Principal Findings:**

*Clonorchis sinensis* eggs were detected in 122 of 183 gallbladder stones based on morphologic characteristics and results from real-time fluorescent PCR. The proportion of pigment stones, cholesterol stones and mixed gallstones in the egg-positive stones was 79.5% (97/122), 3.3% (4/122) and 17.2% (21/122), respectively, while 29.5% (18/61), 31.1% (19/61) and 39.3% (24/61) in the egg-negative stones. The proportion of pigment stone in the *Clonorchis sinensis* egg-positive stones was higher than in egg-negative stones (*P<0.0001*). In the 30 egg-positive stones examined by scanning electron microscopy, dozens or even hundreds of *Clonorchis sinensis* eggs were visible (×400) showing a distinct morphology. Many eggs were wrapped with surrounding particles, and in some, muskmelon wrinkles was seen on the surface of the eggs. Also visible were pieces of texture shed from some of the eggs. Some eggs were depressed or without operculum while most eggs were adhered to or wrapped with amorphous particles or mucoid matter (×3000).

**Conclusion:**

*Clonorchis sinensis* eggs were detected in the gallbladder stones which suggests an association between *Clonorchis sinensis* infection and gallbladder stones formation, especially pigment stones.

## Introduction

Cholecystolithiasis or the presence of gallstones in the gallbladder is a common disease with a mean prevalence rate of 10% based on epidemiological studies [1]. The incidence rate increases with changes in dietary patterns and with the development of an aging population [2]–[6]. In spite of its high incidence rate, the etiology and pathogenesis of gallbladder stones is still not well understood [7]–[12].

Clonorchiasis, also known as liver fluke disease, is an important amphixenosis. It is mainly distributed in East Asia and Southeast Asia, including China, DPR.Korea, the Republic of Korea, Vietnam, and the Philippines [13]. Based on the second national survey on parasitic diseases between 2001 and 2004 in China, the overall *C. sinensis* infection rate of the surveyed population was 0.58% [14], [15]. More surveys at known endemic areas in China found 2.4% people infected with *C. sinensis* [16]. Guangdong had the highest infection rate (16.4%) among provinces, followed by Guangxi (9.8%) [16].

Many studies based on epidemiology and imaging have shown that gallstones are associated with *C. sinensis* infection [17]–[24], many researchers considered that *C. sinensis* eggs may become the nucleus of gallstones and then promote the formation of gallstones, but there has been no direct evidence for the association of *C. sinensis* infection and gallbladder stone formation. We found *C. sinensis* eggs in gallbladder stones by light microscopy incidentally. To test a possible link between *C. sinensis* infection and gallbladder stone formation, we analyzed gallbladder stones by microscopy as well as real-time PCR for the presence of *C. sinensis* adult worm and/or eggs. *C. sinensis* eggs were indeed present in a significant number of gallbladder stones, providing the first direct evidence for an association between *C. sinensis* infection and gallbladder stone formation.

## Materials and Methods

### Ethics Statement

A written informed consent was obtained from all subjects. This research was approved by the Medical Ethics Committee of The Second People’s Hospital of Panyu, Guangzhou.

**Figure 1:**
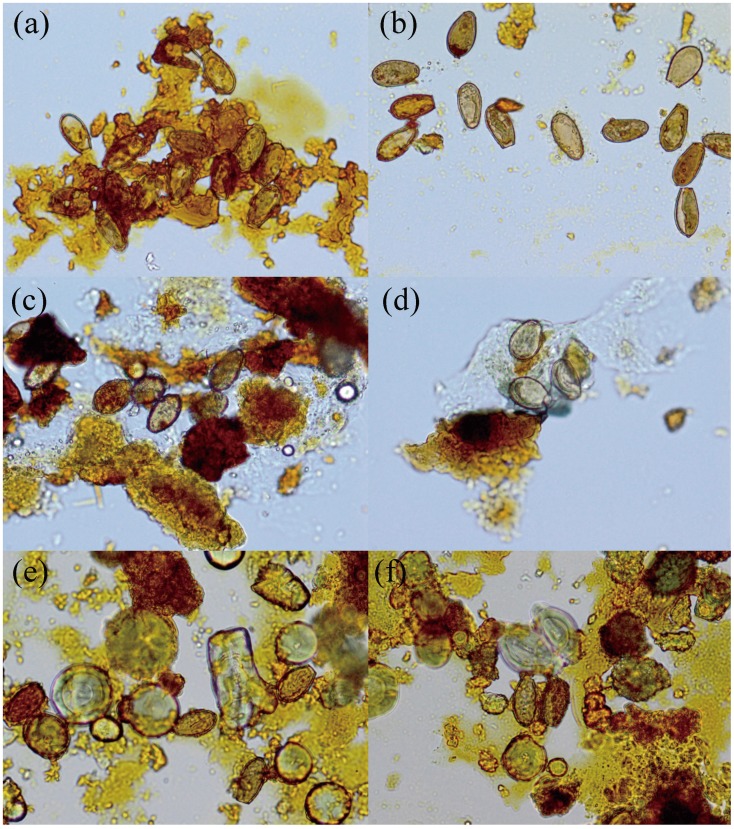
*C. sinensis* eggs in gallbladder stones under a light microscope (original magnification, ×400). **a, b,** eggs are yellow-brown, and wrapped by bilirubinate granules, and parts of the eggs have lost operculum and have a thick shell; **c, d,** eggs are wrapped by bilirubinate granules or mucoid matter, with operculum in the front end and miracidium in the egg; **e, f,** eggs are adhered to or parceled with bilirubinate granules and calcium carbonate crystals, and part of the eggs have lost operculum and have a thick shell.

### Subjects and Specimens

Gallbladder stones from one hundred eighty-three cholecystolithiasis patients that had received gallbladder-preserving cholelithotomies in the Department of General Surgery of The Second People’s Hospital during the period of March 2011 to August 2011 were obtained. All patients were from Guangdong province, and consist of 101 males and 82 females. The mean age was 46.6±12.8 (*Mean* ±*SD*) years old. The BMI of male and female patients was 23.83±3.37 and 23.08±3.97 (*Mean* ±*SD*) respectively. Of all the patients, 49.7% (91/183), 14.2% (26/183), 30.1% (55/183) and 6.0% (11/183) were with moderate weight, underweight, overweight and obesity.

During the operation, no indications such as bile leakage and common bile duct injury occurred. The number of stones from each patient ranged from one to hundreds; single stones and multiple stones was found in 21.9% (40/183) and 78.1% (143/183) of patients respectively. All stones from one patient were counted as one case; all the 183 cases of stones were analyzed in the present study.

**Figure 2:**
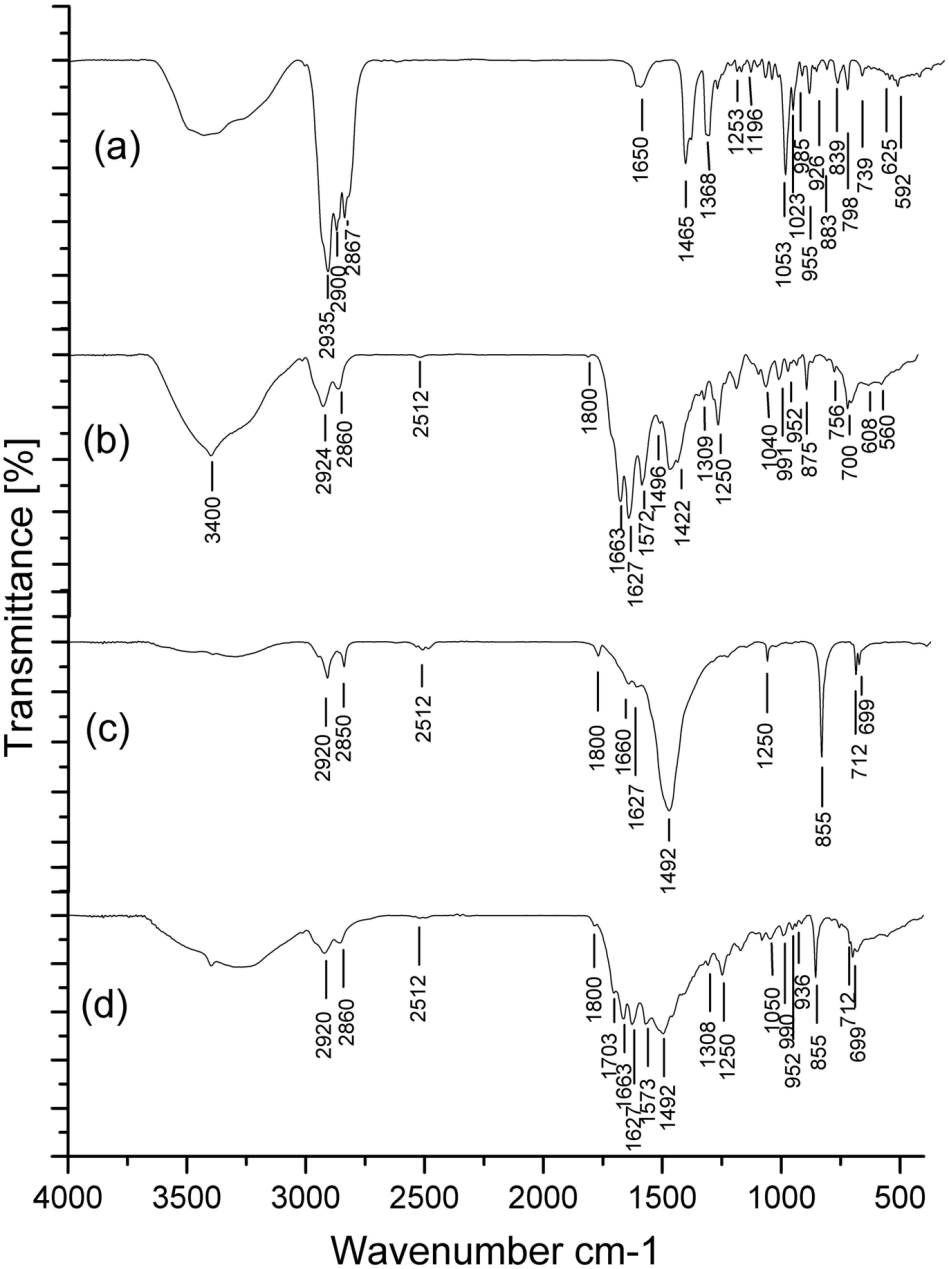
The characteristic spectrum of three types of gallbladder stones. **a,** characteristic spectrum of cholesterol stones; 1465, 1368, 1196, 1135, 1053, 1023, 985, 955, 883, 839, 798, 739, 592 are the peaks of cholesterol, 1650, 1253, 1196, 1109, 985, 926, 625 are the peaks of bilirubin, so the main component is cholesterol, containing a small amount of bilirubin; **b,** characteristic spectrum of pigment stones; 1663, 1627, 1572, 1496, 1309, 1250, 1040, 991, 937, 849, 756, 700 are the peaks of the bilirubin and bilirubinate, 1050, 991, 952, 849 are the peaks of cholesterol, 1800, 1422, 875 are the peaks of calcium carbonate, 1627, 1572, 1422 are peaks of calcium stearate, 1040, 608, 568 are the peaks of hydroxyapatite, so the main components are bilirubin and bilirubinate, containing a small amount of calcium stearate, calcium carbonate, hydroxyapatite and cholesterol; **c, d,** spectrum of the outer layer and core of mixed stones; **c,** the outer layer of the mixed stones, 1800, 1492, 855, 712, 699 are the peaks of calcium carbonate, 1660, 1627, 1250 are the peaks of bilirubin and bilirubinate, so the main component of the outer layer is calcium carbonate, containing a small amount of bilirubin and bilirubinate, **d,** the core of the mixed stones, 1703, 1663, 1627, 1573, 1308, 1250, 1105, 1050, 990, 936, 756, 699, 555, 434 are the peaks of bilirubin and bilirubinate, 1800, 1492, 855, 712, 699 are the peaks of calcium carbonate, 1050, 991, 952 are the peaks of cholesterol, 1627, 1573, 1105, 712 are the peaks of calcium stearate, so the main components of the core are bilirubin and bilirubinate, containing calcium carbonate, cholesterol and small amounts of calcium stearate.

**Figure 3:**
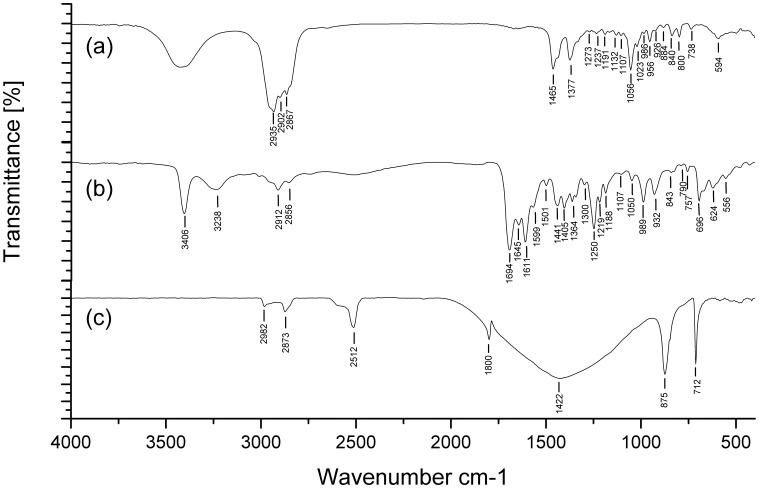
The spectrum of the standard controls. a, spectrum of cholesterol standard; **b,** spectrum of bilirubin standard; **c,** spectrum of calcium carbonate standard.

### Microscopic Examination of Gallbladder Stones (Chinese National Inventive Patent Number: ZL201010123552.3)

Gallbladder stones were washed twice with distilled water, and then dried at 60°C for 12 hours. Subsequently, stones were split, and about 10 mg of each layer was weighed if the hierarchical structure of the profile was clear. Otherwise, about 10 mg was weighed directly, put in a mortar and 300 µl of 0.9% NaCl was added. The stones were ground thoroughly and then filtered with a 260 mesh nylon yarn. Each filtrate was then smeared onto 3–4 labeled slides and scored for the presence or absence of *C. sinensis* eggs under an Olympus System Microscope (BX51, Japan).

**Table 1:** The detection of eggs in three types of gallbladder stones with light microscopy.

Type of stones	Pos. No.	Neg. no.	total	Detectionrate	*P value*
**Pigment stones**	97	18	115	84.3%	*<0.0001*
**cholesterol stones**	4	19	23	17.4%	
**mixed stones**	21	24	45	46.7%	
**Total**	122	61	183	66.7%	

### Detection of *C. sinensis* DNA in the Gallbladder Stones by Real-time Fluorescent PCR

Gallbladder stones that were scored positive or negative for eggs (thirty each) based on morphology were chosen randomly from the pool of stone samples, for confirmation of the presence of *C. sinensis* DNA using real-time fluorescent PCR. DNA from a *C. sinensis* adult was used as a positive control.

#### Extraction of DNA

Adult of *C. sinensis*: The adult worm of *C. sinensis* obtained from clinical patients was first washed twice with 0.9% NaCl, then dried on the filter paper and ground thoroughly in a mortar by adding liquid nitrogen constantly. Only a single specimen was used and DNA was extracted using the DNeasy Blood and Tissue Kit (Qiagen) according to manufacturer’s instructions. Briefly, the grinding powder was suspended into 180 µl of ATL buffer, and then 20 µl proteinase-K was added and incubated at 58°C for 4 h with brief vortexing every 30 min. Thereafter, 200 µl AL buffer and 200 µl absolute alcohol was added successively and mixed by vortexing for 15–20 s. Finally, the genomic DNA was collected using DNeasy Mini spin column and eluted in 100 µl elution buffer (AE) and stored at −20°C until use. Gallbladder stones: For mechanical breaking, 20 mg of the stones were weighed and frozen in liquid nitrogen gas for 10 min, and then ground thoroughly in a mortar. After adding 1 ml 0.9% NaCl, the suspension was transferred to a new 2.0 Ep tube and centrifuged at 12000 rpm for 10 min. The pellet was treated and used for DNA extraction with a DNeasy Blood Tissue Kit (Cat.No. 69504, Qiagen, Germany) in accordance with the manufacturer’s instructions.

**Figure 4:**
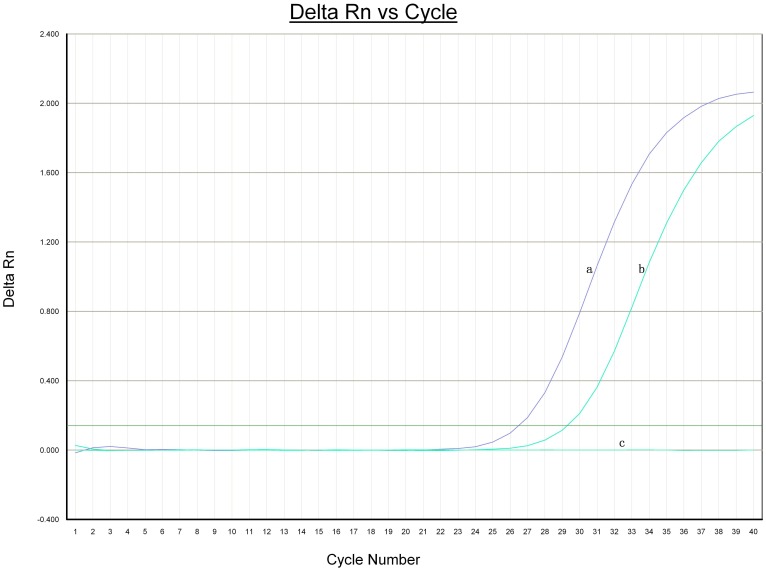
Gene identification of eggs in gallbladder stones. **a,** DNA of adults; **b,** DNA of egg-positive gallbladder stones; **c,** DNA of egg-negative gallbladder stones. The cycle threshold (Ct) for *C. sinensis* adult and egg-positive gallbladder stone was 26.49 and 29.21 respectively.

#### Fluorescent PCR

The primers and Taqman probe were designed to detect the cytochrome C oxidase subunit 1 gene of *C. sinensis* (GenBank: FJ965388.1) using the Beacon Designer-v 7.51 software. It was then submitted to the BLAST program of NCBI for specific analysis. These primers Cs-F (5′-GGTTTGGTATGATTAGTCACATTTG-3′) and Cs-R.

(5′-ACCACCCTACCCAGACAAAC-3′) amplify a 121 bp fragment of the COI sequence. The minor-groove-binding TaqMan-probe Cs-P.

**Figure 5:**
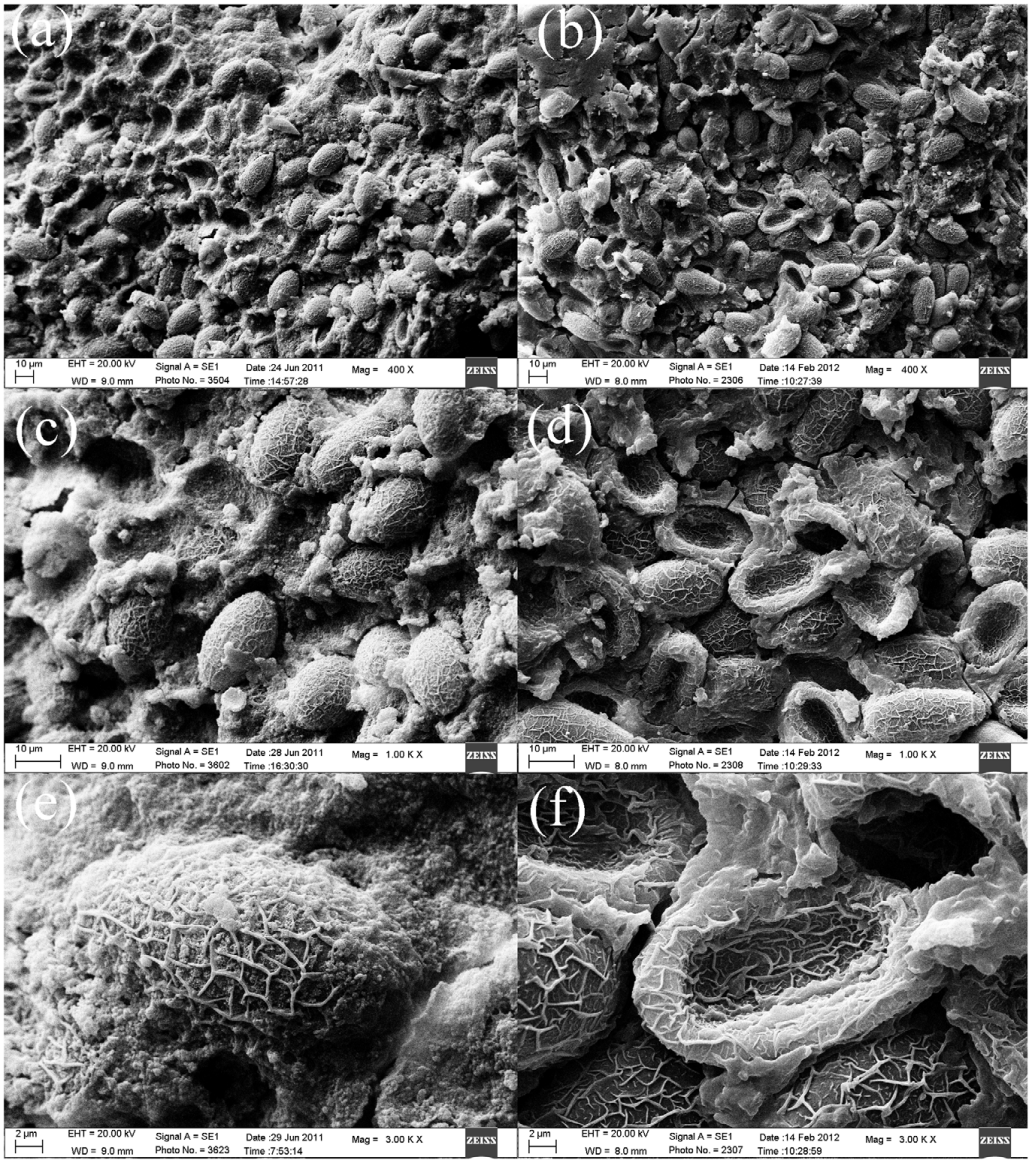
The egg-positive stones under SEM. **a, b,** dozens or even hundreds of *C. sinensis* eggs were visible (original magnification, ×400); **c, d,** a dozen or even dozens of *C. sinensis* eggs were adhered to or wrapped with surrounding particles (original magnification, ×1000); **e, f,** muskmelon wrinkles was observed on the surface of the eggs, part of the eggs had depressed surface and particulate matter adhered on the surface and surrounding the eggs (original magnification, ×3000).

**Figure 6:**
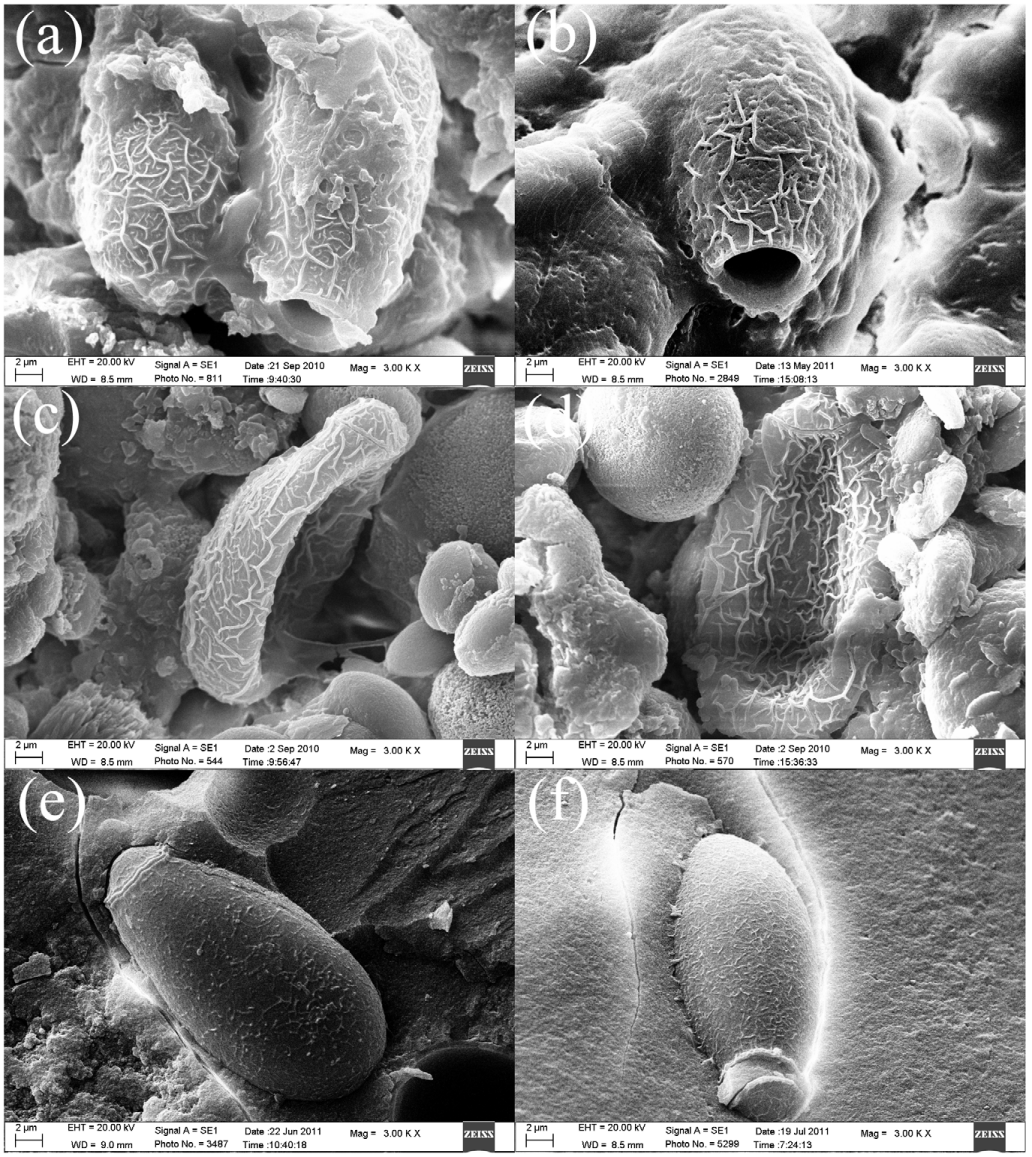
*C. sinensis* eggs with morphologic changes under SEM (original magnification, ×**3000). a, b,** eggs were without operculum, and adhered to or wrapped with bilirubinate granules or mucoid matter; **c, d,** eggs were depressed, and adhered to bilirubinate granules, calcium carbonate crystals or mucoid matter; **e, f,** eggs had a shedding texture or their operculum had split from the shell.

(5′-JOE-AGCAAACATAGCCAACACCAAGCCC-BHQ1-3′) was used to detect the *C. sinensis* specific product. The real-time PCR assay was run in a total reaction volume of 50 µl. The final concentration of the reaction solution included 0.2 µM of both forward and reverse primers, 0.2 µM of the TaqMan probe, 25 µl of *Premix Ex Taq*™ (Takara), 1 µl ROX buffer and 2 µl templates. The real-time PCR cycling parameters were set as follows: initial 95°C for 30 s, then 45 cycles of 15 s at 95°C and 31 s at 60°C. PCR amplification, detection, and data analysis were performed with an ABI 7300 fluorescence quantitative PCR instrument (Applied Biosystems, Foster City, CA, USA).

### Composition Analysis of Gallbladder Stones by Fourier Transform Infrared (FTIR) Spectroscopy

Gallbladder stones samples were analyzed with a Bruker (TENSOR27, Germany) FTIR spectrometer in the frequency range 400–4000 cm-1 at 4 cm-1 resolution. For each measurement, 2 mg of finely powdered stone sample mixed with 300 mg KBr was used to make discs. Control spectrums were acquired from standard substances, which were of high quality (99% pure) and purchased from Sigma Chemical Company (St. Louis, MO, USA). Through comparisons of the spectra between gallbladder stones and standard controls, gallbladder stones were grouped as cholesterol stones, pigment stones or mixed stones [25], [26].

### Observation of Gallbladder Stones with Scanning Electron Microscopy (SEM)

Thirty egg-positive gallbladder stones were chosen for SEM analysis. Stones were washed thoroughly with distilled water and dried at 60°C. Then, they were split and one piece was taken from each layer if the hierarchical structure of the profile was clear; otherwise, one piece from the surface and one piece from the core were taken and then fixed on the sample table and dried at 60°C overnight. Then, the stones were sputter-coated with gold (ETD-2000, China) and observed under a ZEISS (EVO LS10, Germany) SEM and photographed. The EHT was 20 kv.

### Statistical Analysis

Age was analyzed as *mean* ±*SE*, the detection rate was analyzed using a chi square test and the partitions of the chi square method were used for multiple comparisons using *SPSS v.11.5* software. *P<0.05* was regarded as statistically significant.

## Results

### *C. sinensis* Eggs were Detected in the Gallbladder Stones using Light Microscopy and Egg-positive Gallbladder Stones were Mainly Pigment Stones

*C. sinensis* eggs are yellow-brown in color, resemble sesame seeds, have a thick shell and a small operculum in the front end, shouldering in the junction of the shell and operculum, with occasional visible small protrusions in the obtuse posterior end. Through microscopic examination, *C. sinensis* eggs were found in 122 of 183 gallbladder stones, providing a detection rate of 66.7%. Meanwhile, large portions of the eggs were adhered to or wrapped by bilirubinate granules and/or mucoid matter. Based on these observations, eggs in the gallbladder stones were preliminarily judged as *C. sinensis* eggs (as shown in Figure 1).

To determine the main composition of the stones, we analyzed the stones using FTIR spectroscopy. Results showed that the 183 gallbladder stones were composed of 115 pigment stones, 45 mixed stones and 23 cholesterol stones. The characteristic spectrum of the three types of stones is shown in Figure 2 and the spectrum of the standard controls is shown in Figure 3. The FTIR spectroscopy analysis revealed that 122 egg-positive gallbladder stones were composed of 97 pigment stones, 21 mixed stones and 4 cholesterol stones, while the 61 egg-negative stones were composed of 18 pigment stones, 24 mixed stones and 19 cholesterol stones. The proportion of pigment stone in the egg-positive gallbladder stones was higher than those in egg-negative stones (*P<0.0001*); in other words, egg-positive gallbladder stones were mainly pigment stones, as shown in Table 1.

### *C. sinensis* Eggs in the Gallbladder Stones were Further Confirmed by Real-time Fluorescent PCR

In order to confirm that the eggs detected in the gallbladder stones were *C. sinensis* eggs, we analyzed the gallbladder stones for the presence of *C. sinensis* DNA using real-time fluorescent PCR. A positive amplification curve emerged in the DNA of the positive control and egg-positive stones (Figure 4), but no positive amplification curve (a straight line) in the DNA of egg-negative stones. These results confirm that the eggs detected in the gallbladder stones were *C. sinensis* eggs, and it was consistent with that of the microscopy.

### Many *C. sinensis* Eggs were Discovered in the Gallbladder Stones Under SEM

To investigate the association between *C. sinensis* eggs and gallbladder stones more accurately, while preserving the ultrastructural histology, we analyzed 30 stones that scored positive for *C. sinensis* using previous methods for the presence of *C. sinensis* eggs using SEM. To our surprise, dozens or even hundreds, in some cases, of *C. sinensis* eggs were visible in the stones (Figure 5, original magnification, ×400). Several *C. sinensis* eggs were adhered to or wrapped with surrounding particles and/or mucoid matter (original magnification, ×1000). Muskmelon wrinkles was seen on the surface of the eggs, which interrupted the edge of the operculum. The junction of the shell and operculum was loose and appeared shouldering. Part of the eggs had visible small protrusions in the posterior end and part of the eggs were depressed or without operculum. Pieces of texture shed from some eggs, and amorphous particles or mucoid matter were adhered to the surface, surrounding the eggs as shown in Figure 6 (original magnification, ×3000).

## Discussion

Clonorchiasis, a zoonotic disease, is caused by *C. sinensis* that parasitizes the human intrahepatic bile duct. Humans are often infected with *C. sinensis* due to the consumption of undercooked freshwater fish or shrimp containing metacercariae [27]–[29]. Clonorchiasis can produce complications including cholangitis, gallstones, cholangiohepatitis, recurrent pyogenic cholangitis, cirrhosis and cholangiocarcinoma [13], [14], [17], [30]–[38]. In addition, *C. sinensis* infection may promote gallstone formation as determined previously using radiological examination, ultrasonic inspection, serologic examination and microscopic examination of feces and bile [17]–[21], [39], [40]. Furthermore, other researchers have confirmed that *C. sinensis* infection is involved in the formation of bile duct stones based on the presence of *C. sinensis* eggs or genes in the bile duct stones [21], [22]. Nevertheless, there have been few reports investigating the association of *C. sinensis* infection and cholecystolithiasis through analysis of gallbladder stones.

In the present study, we detected *C. sinensis* eggs in gallbladder stones from 66.7% of the patients with cholecystolithiasis through light microscopy. In other words, 66.7% patients with cholecystolithiasis are infected with *C. sinensis*. Furthermore, the results of microscopic examination revealed that a large portion of the eggs were adhered to or wrapped by bilirubinate granules, mucoid matter and calcium carbonate crystals, which indicate that *C. sinensis* eggs might be the important nuclear factor involved in the formation of the gallbladder stones, especially pigment gallbladder stones. Coincidentally, results from FTIR spectroscopy analysis showed that the egg-positive stones were mainly pigment stones. However, some researchers have proposed that there is no relationship between *C. sinensis* infection and cholecystolithiasis from a comparison of patients with and without *C. sinensis* infection [18]–[20]. We think that the two opposing conclusions may be due to the difference in the types of samples analyzed and in the experimental methods employed (e.g. diagnosis of *C. sinensis* infection). Remarkably, the egg-positive stones were mainly pigment stones. These results suggest that although *C. sinensis* infection may not necessary lead to increased incidence of cholecystolithiasis [18]–[20], it is positively associated with high proportion of pigment stones. It remains to be investigated whether *C. sinensis* infection is the etiological factor for cholecystolithiasis.

Due to the subjectivity inherent in morphological analysis by light microscopy, we used real-time fluorescence PCR to detect the presence of *C. sinensis* eggs. The cytochrome C oxidase subunit 1 gene, a mitochondrial gene in *C. sinensis* present in hundreds or thousands of copies per cell, is more highly conserved than the ITS-2 sequences of *C. sinensis* and thus may be a good target for the development and evaluation with real-time PCR [41], [42]. A real-time PCR assay based on the cytochrome coxidase subunit 1 gene of *C. sinensis* was previously used to successfully detect *C. sinensis* DNA in stool samples of rats [43], [44]. Therefore, in the present study, we chose the cytochrome C oxidase subunit 1 gene as the target gene for real-time PCR detection, and the results confirmed that the eggs in the gallbladder stones were indeed *C. sinensis* eggs.

To further explore the relationship between *C. sinensis* eggs and gallbladder stones formation without destroying the original structure of the stones, we analyzed the stones by SEM. SEM analysis revealed the presence of a large number of bilirubinate granules and a small amount of calcium carbonate crystals in the egg-positive gallbladder stones. Meanwhile, many of the *C. sinensis* eggs with a textured surface were discovered in gallbladder stones, and many tiny bilirubinate granules were adhered to the muskmelon wrinkles. Some eggs were wrapped with mucoid matter, while some were depressed or with thickened shell and/or without operculum. Other eggs displayed a shedding texture. The morphologic changes and diversity in the eggs might result from the extended periods that they had existed in the stones, or from nutritional deficiencies, calcification, nucleation and other factors. Based on the morphological study results, we conclude a possible mechanism for the formation of gallbladder stones involving *C. sinensis* eggs. The inherent texture of *C. sinensis* eggs is cancellate and uneven, and it is easy for particles and crystals to adhere to its surface. Also, the eddy effect of the bile during gallbladder contraction may lead to the deposition and aggregation of many eggs. Moreover, because of the intermediary role of mucus (mucin) secreted by the gallbladder and the stimulation of *C. sinensis* eggs and/or worms metabolic product, *C. sinensis* eggs adhere to or become enveloped by surrounding particles, mucoid matter and crystals. This becomes the nucleus of the gallbladder stone, and consolidation of these nuclei occurs to promote the formation of gallbladder stones.

### Conclusion

Our data indicate that *C. sinensis* eggs may directly participate in the formation of gallbladder stones, especially pigment gallbladder stones. Results reveal a possible association between *C. sinensis* eggs and formation of gallbladder stones, which contributes to our knowledge on the etiology of clonorchiasis and gallbladder stone formation. Also, our results suggest that blocking water pollution, preventing the intake of water and food contaminated by *C. sinensis*, changing eating habits (eating raw freshwater fish or shrimp), or applying anti-*C. sinensis* treatment in patients with *C. sinensis* eggs would reduce not only the prevalence of clonorchiasis but also the incidence of pigment gallstones in the epidemic areas.
